# Bioinformatic identification of CD8+ T cell activation mediated by key genes in fecal microbiota transplantation for irritable bowel syndrome

**DOI:** 10.1371/journal.pone.0351574

**Published:** 2026-06-16

**Authors:** Ying Fei, Ming-Yi Gao, Nan Qiao, Jia Hu, Ling He, Jiao-Li Zhou, Ning-Ning Zheng, Ting-Ting Liu

**Affiliations:** 1 Department of Gastroenterology, YingTan Chinese Medicine Hospital, Yingtan, Jiangxi, China; 2 Graduate College, Jiangxi University of Chinese Medicine, Nanchang, Jiangxi, China; 3 Department of Student Affairs, Jiangxi Flight University, Nanchang, Jiangxi, China; 4 Department of Gastroenterology, The Affiliated Hospital of Jiangxi University of Chinese Medicine, Nanchang, Jiangxi, China; Pennsylvania State University Hershey Medical Center, UNITED STATES OF AMERICA

## Abstract

**Background:**

The effect of fecal microbiota transplantation (FMT) in treating irritable bowel syndrome (IBS) may be attributed to the modulation of CD8 + T cells. This study aims to identify FMT-mediated key genes to explore the underlying mechanism.

**Methods:**

Transcriptomic datasets GSE138297 (colonic biopsies from 8 IBS patients pre- and post-FMT) and GSE134649 (single-cell data from 3 healthy colon tissues) were obtained from GEO during December 2023–December 2024. Key genes were identified by intersecting differentially expressed genes (DEGs) and the most relevant co-expression module derived from weighted correlation network analysis. Functional enrichment, gene set enrichment analysis, immune infiltration profiling via TIMER 2.0, single-cell annotation using PanglaoDB and Seurat, and drug–gene interaction screening from DrugBank were conducted to decipher the regulatory mechanisms.

**Results:**

Ten key genes were identified through integration of DEGs and the MEgreen module. Functional analyses revealed significant involvement in the positive regulation of CD8 + T cells activation. Immune infiltration assessment demonstrated a marked increase in CD8 + T cells abundance post-FMT. Single-cell data indicated predominant expression of LILRB1, P2RY13, CLEC10A, and CLEC12A in dendritic cells, and LILRB1, PIPOX, and CLEC11A were annotated within CD8 + T cells clusters in healthy colonic tissue. Nine (database-derived and speculative) drugs targeting seven key genes were identified, most implicated in the management of IBS symptoms or immunomodulation.

**Conclusion:**

An association between key gene regulation and CD8 + T cell-related immunoregulation is correlated with the therapeutic effect of FMT in IBS.

## Introduction

The global prevalence of irritable bowel syndrome (IBS) has reached 5–10% [1], and is still rising. Chronic recurrence frequently precipitates multiple complications, including colitis, metabolic imbalances and psychological disorders, etc [2,3]. These have a serious impact on quality of life and even hide malignant tumors [4]. Fecal microbiota transplantation (FMT) has emerged as a promising strategy for treating IBS [5,6]. It was reported that the three-year response rate for FMT in patients with IBS was 71.8%, compared with 27.0% for the placebo group [7]. However, several challenges of FMT have been inevitable from the unsatisfactory relapse rate, the response variation among patients, and the lack of an ideal schedule with a consensus standard [8]. Therefore identifying key molecules that drive FMT and further deciphering the biological mechanisms by which key molecules support FMT is imminent to optimize IBS treatment.

The most studied biological mechanism of FMT is immune regulation [9,10], and CD8 + T cells activation is increasingly recognized and implicated. In a study by Yu et al., FMT reversed the disruption of the gut microbiota in mice with colorectal cancer. This resulted in the extensive infiltration of CD8 + T cells, which in turn led to the direct clearance of tumor cells [11]. When it comes to IBS, the increased dendritic cells (DCs) in the colon fueled CD8 + T cells to secrete high levels of IL-9, leading to mast cell activation and subsequent visceral hypersensitivity [12]. Although CD8 + T cells activation plays a pivotal role in the treatment of IBS with FMT, the key molecules that initiate CD8 + T cells activation by the microbiota remain to be elucidated. Consequently, the comprehensive biological response of FMT is deficient in comprehensive analysis and mutual validation.

The rapid development of bioinformatics has provided a fresh perspective for deciphering biological mechanisms that are lacking in randomized controlled trials (RCT). A typical case is the identification of key genes most pertinent for occurrence and prognosis from Gene Expression Omnibus (GEO) datasets, derivation of dominant factors from biological processes (BP) and pathways based on key genes, and multidimensional validation of their supporting biological mechanism in other datasets with multi-omics analyses. Utilising this strategy, Gu et al.[13] identified nine key genes that regulate iron metabolism, thereby facilitating a more comprehensive understanding of the prognosis and pharmacological treatment of Alzheimer's disease.

**Fig 1:**
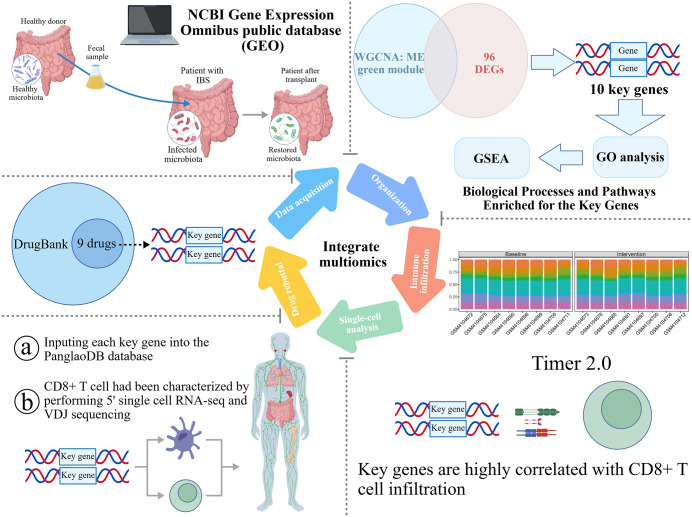
The workflow of the analyses.

In this study, we aimed to decipher the regulatory mechanism of CD8 + T cells activation during FMT in the treatment of IBS based on key genes and validate it as a relatively complete bioinformatics chain in multiple dimensions, including transcriptomic information enrichment, immune infiltration, single-cell expression, and drug development. We also sought to provide a proof-of-concept paradigm for similar mechanistic studies. The workflow of this study is shown in Fig 1.

This study integrated transcriptomic and single-cell datasets from the GEO database to identify key genes mediating FMT-induced CD8 + T cell activation in IBS.

## Materials and methods

### Data acquisition

Gene expression data were downloaded from GSE138297 and GSE134649 of GEO. This study was conducted between December 2023 and December 2024. GSE138297 included 16 colonic biopsy samples from eight IBS patients (eight baseline samples and eight post-FMT samples). GSE134649 included single-cell clusters isolated from colonic biopsies of three healthy donors.

### Key genes dig

#### Weighted Correlation Network Analysis (WGCNA).

Gene co-expression networks were constructed [14]. First, a similarity matrix was constructed. This was then converted into an adjacency matrix by selecting an appropriate threshold. A topological overlap matrix was then generated to group genes with similar expression patterns into different modules. Pearson correlation was used to evaluate module-trait associations, and the module with the highest absolute value of the positive correlation coefficient was finally selected for further analysis.

#### Differentially Expressed Genes (DEGs) identification.

Differential expression analysis was conducted between the baseline and intervention samples using the “limma” package in R software. Student’s t-test was used for differential analysis, genes with *p* < 0.05, and abs (logFC) > 0.5 were identified as DEGs. Volcano plot of DEGs was generated using the “ggplot2” package in R software.

The “Venn Diagram” package in R software was used to obtain the intersection genes of DEGs and the module by WGCNA. Differences in the expression of key genes between baseline and intervention samples were represented by violin plots (*p* < 0.05).

### Analyses of biological mechanisms based on key genes

#### Functional enrichment analysis.

The BP of Gene Ontology (GO) were incorporated and depicted in the network diagram utilizing the “GO plot” package in R software. The functions of these genes were analyzed through Gene Set Enrichment Analysis (GSEA). The results were visualized utilizing the R package “enrich plot” Functional annotations were conducted using the R package “cluster Profiler” applying a screening criterion of p-adjusted < 0.05.

#### Validation of immune infiltration.

TIMER 2.0 was employed to evaluate immune cell infiltration within the microenvironment [15]. The relative proportions and abundance of various immune cell types in each sample were quantified. In particular, an association analysis was conducted based on key genes.

### Validation of single-cell expression

#### Cell annotation from PanglaoDB.

Each key gene was entered into the PanglaoDB database to retrieve the cell type and the number of cell clusters that were annotated with the key genes [16]. The results were presented as a “bar plot.”

#### Cell annotation by single-cell sequencing.

In GSE134649, Cells were characterized by performing 5’ single-cell RNA-seq and VDJ sequencing. Key genes-oriented cell clusters were annotated by TSNA analysis with “seurat” package.

### Validation of drug development

Drugs targeted by key genes were searched using DrugBank [17]. Drug-gene interaction may validate the biological mechanism supported by key genes in the treatment of IBS from the perspective of drug development.

### Ethics approval and consent to participate

This study analyzed publicly available, anonymized GEO datasets. No human or animal subjects were involved; ethical approval was not applicable.

## Results

### Key genes identification

**Fig 2:**
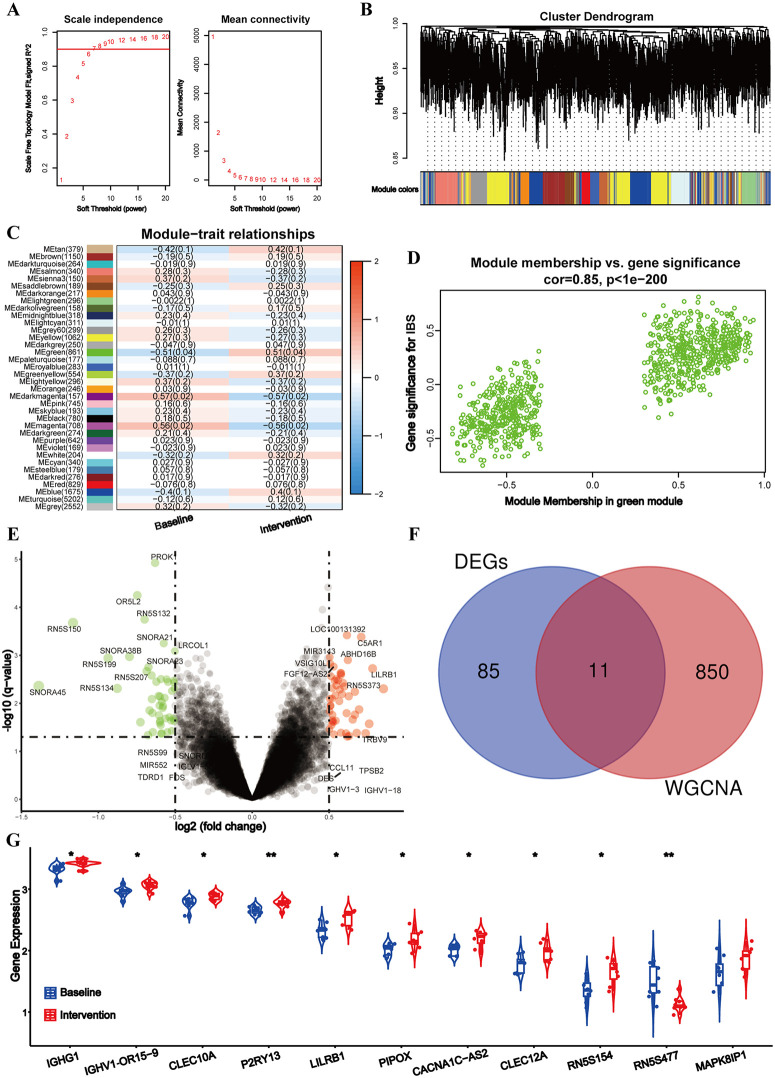
Results of the WGCNA and key genes between baseline and intervention samples. **(A)** The corresponding scale-free topological model fit indices and mean connectivity values at different threshold powers. **(B)** Cluster dendrogram of genes. **(C)** Correlations between different modules and clinical traits, red = positive correlation, blue = negative correlation. **(D)** Correlation of module membership and gene significance in the green module (Pearson correlation, cor = 0.85, *P* < 1e-200). **(E)** Volcano plot of DEGs (filtered by *P* < 0.05 and |logFC| > 0.5); red = upregulated genes, green = downregulated genes. **(F)** 11 intersecting genes were obtained by Venn between DEGs and MEgreen module from WGCNA. **(G)** Expression of key genes in the baseline and intervention groups (Student’s t-test, *: *P* < 0.05, **: *P* < 0.001).

The expression profiles of 16 samples and 22,728 genes from the GSE138297 dataset were utilized to construct a weighted gene co-expression network. When the threshold power was set to seven, the scale independence reached 0.905, and the average connection value was 51.1 (Fig 2A). When the cut height was set to 0.4 and the minimum module size was set to 100, 35 different co-expression modules were obtained by dynamic tree cutting (Fig 2B). The MEgreen module, consisting of 861 genes, had the strongest positive correlation coefficient with the phenotype (r = 0.51; *p* = 0.04; Fig 2C). There was a strong correlation between module membership (MM) and gene significance (GS) (cor = 0.85; *p* < le-200; Fig 2D). From eight baseline and eight intervention samples, 96 DEGs were obtained and presented as volcano plots (Fig 2E). By intersection analysis between the MEgreen module genes and DEGs (Fig 2E), 11 genes were mapped (Fig 2F), ten of which were presented as key genes with significant differences (*p* < 0.05, Student’s t-test) in violin plots (Fig 2G), while *MAPK8IP1* was excluded (*p* = 0.071).

### Functional enrichment analyses based on key genes

**Fig 3:**
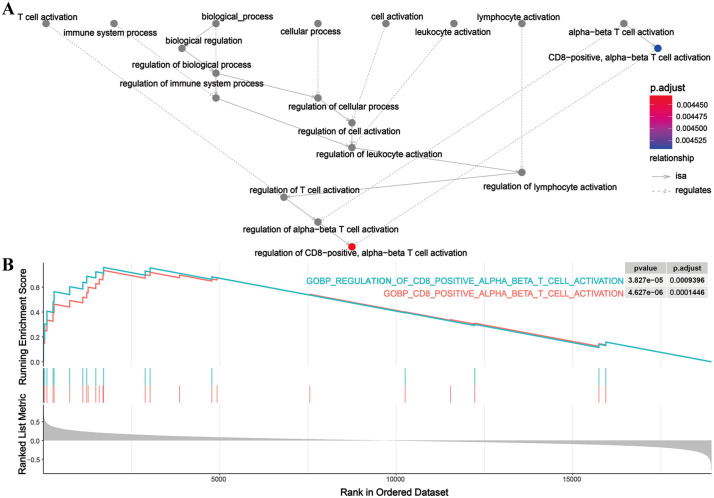
The biological process of orienting CD8 + T cells activation. **(A)** The process was elucidated through the mediation of key genes (*p*-adjusted < 0.05). **(B)** GSEA indicated that CD8 + T cells activation is positively associated with specific biological pathways.

GO enrichment analysis was used to derive the BP based on key genes, and ultimately focused on the regulation of CD8 + T cells activation (Fig 3A). GSEA supported a significant and positive correlation trend in the gene sets (Fig 3B). These results indicated an association between key gene regulation and CD8 + T cell activation following FMT in IBS. This finding will be validated by further analyses.

### Validation of immune infiltration for CD8 + T cells and key genes

**Fig 4:**
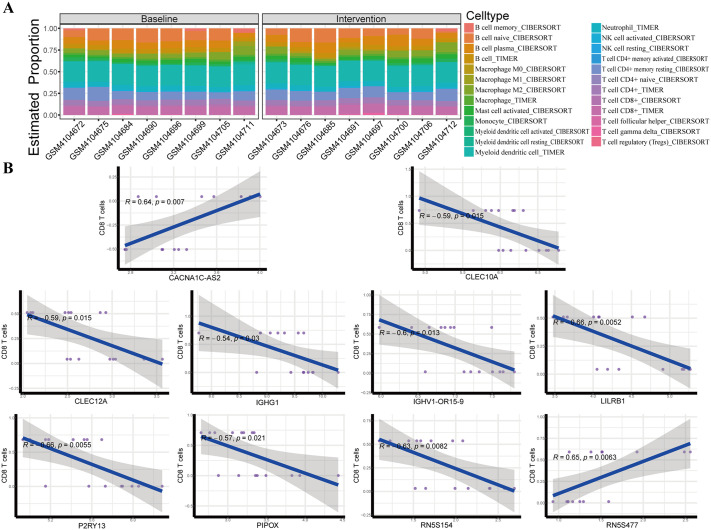
Immune infiltration comparison between baseline and intervention samples. **(A)** The relative proportions of 25 immune cell types were quantified by TIMER 2.0 between baseline and intervention samples (Student’s t-test). **(B)** Significant correlations between key gene expressions and CD8 + T cells populations (Pearson correlation, *R* > 0.5, *P* < 0.05).

The proportion of 25 immune cell types in the two groups was estimated, revealing a significant increase in the proportion of CD8 + T cells in the intervention group (Fig 4A). The correlation between key genes and CD8 + T cells was analyzed individually, with each key gene showing a significant positive association with CD8 + T cells (R > 0.5, p < 0.05; Fig 4B).

### Validation of Single-cell expression between key genes and CD8 + T cells

**Fig 5:**
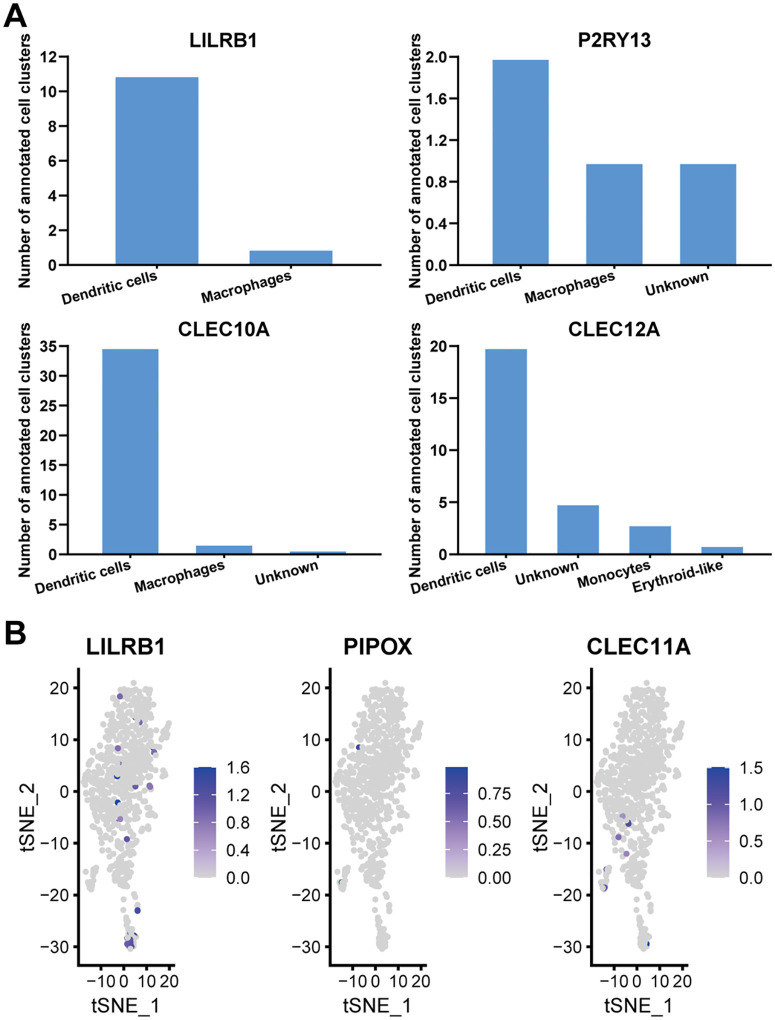
Single-cell expression between key genes and CD8 + T cells. **(A)** Bar plot of cell clusters (Y-axis) and cell types (X-axis) where the gene was expressed based on PanglaoDB. **(B)** Expression and distribution of key genes mapping CD8 + T cells in colonic biopsies of three healthy donors.

Single-cell expression data of six key genes were obtained from PanglaoDB. Among them, *LILRB1*, *P2RY13*, *CLEC10A*, and *CLEC12A* were predominantly annotated as DCs, which activate the initial T cells and play a crucial role in CD8 + T cells activation (Fig 5A).

In the annotation of CD8 + T cells clusters from colonic biopsies of three healthy donors in GSE134649, the expression of *LILRB1*, *PIPOX*, and *CLEC11A* (a member of the *CTL/CTLD* superfamily along with *CLEC10A/CLEC12A*) was obtained and presented with a feature plot (Fig 5B).

### Validation of drug development from the DrugBank Database

**Fig 6:**
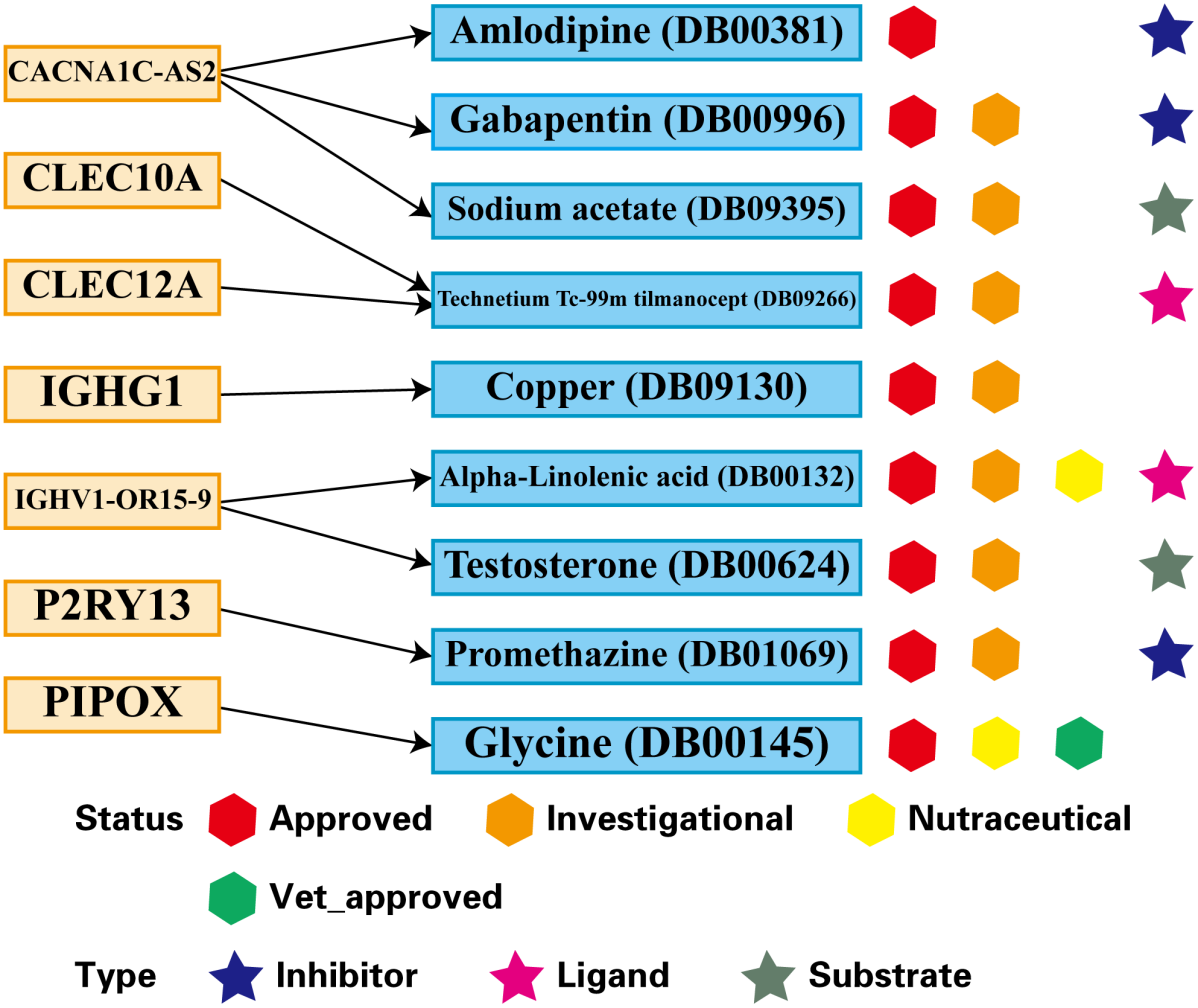
Nine drugs targeted by seven key genes were derived from the DrugBank database.

Nine drugs targeted by seven key genes were derived from the DrugBank database. These drugs were all approved, with seven of them being investigational drugs and the others classified as nutraceuticals. Among these compounds, amlodipine (DB00381) and gabapentin (DB00996) were identified as inhibitors of CACNA1C-AS2. Amlodipine is commonly prescribed for the treatment of hypertensive diseases, while gabapentin functions as an antiepileptic agent. Sodium acetate (DB09395), a substrate of *CACNA1C-AS2*, is used to rehydrate the body and regulate electrolyte balance. Technetium Tc-99m tilmanocept (DB09266), a ligand of *CLEC10A* and *CLEC12A*, is a synthetic radiotracer. Copper (DB09130) is the third most abundant trace element in the human body. Alpha-linolenic acid (DB00132), a ligand for IGHV1-OR15–9, has been employed as an adjunctive therapy for hyperlipidemia and chronic hepatitis. Testosterone (DB00624), a substrate of IGHV1-OR15–9, is indicated for the management of testosterone deficiency and hypogonadism. Promethazine (DB01069), an inhibitor of *P2RY13*, is used to relieve smooth muscle spasms as well as for its antiemetic, anti-motion sickness, and sedative-hypnotic effects. Glycine (DB00145) is used as an amino acid supplement (Fig 6). All drugs are theoretically relevant to IBS management or CD8 + T cell regulation.

All identified drugs are potentially relevant to IBS management or CD8 + T cell regulation. Drug statuses, including approved, investigational, nutraceutical, and vet-approved, are indicated by colored hexagons. Drug types, including inhibitor, ligand, and substrate, are indicated by colored pentagons.

## Discussion

Current investigations into FMT for IBS predominantly emphasize RCTs. However, this study indicated an association between FMT and CD8 + T cell activation in treating IBS from the perspective of key genes, which had not been reported in similar studies. Based on key genes, this CD8 + T cells-oriented regulation was validated and comprehended in a panoramic manner from a multidimensional biological information chain. The results of this study not only conformed to the current mainstream view that immunoregulation plays a key role in FMT treatment [9,10], but also highlighted CD8 + T cells activation, which may have been overlooked in previous IBS studies.

Numerous investigations have concentrated on the identification of DEGs, yet have ignored the correlation among genes. WGCNA is a better strategy for categorizing gene modules with highly correlated expression properties, summarizing the interconnections between the modules and their associations with phenotypes, and identifying potential biokey genes or therapeutic targets [14]. We performed WGCNA to obtain a set of synergistically varied genes, which helped to study the interactions between genes at a holistic level. The MEgreen module was positively and most highly correlated with the modules and phenotypes of MM and GS (cor = 0.85; p < 1e-200; Fig 2D). Therefore, the intersection of DEGs and the MEgreen module was selected to identify key genes.

In BP, the mediation within the gene association network centered on pivotal genes ultimately facilitated the positive regulation of CD8 + T cells activation. Subsequent GSEA supported this general trend in the gene sets. This method of mining key genes to derive biological mechanisms provides grounds for a proof-of-concept of the scientific hypotheses: The key gene regulation linked to FMT is associated with CD8 + T cell activation in the treatment of IBS.

In the validation of immune cell infiltration, all pivotal genes demonstrated significant correlations with CD8 + T cells. *CACNA1C-AS2* plays a role in the immune microenvironment by regulating CD8 + T cells [18]. *CLEC10A* expression correlated with the level of CD8 + T cells infiltration [19]. *CLEC12A* inhibits activation signals, fine-tunes immunity, prevents harmful inflammation [20], and is most prominent in colitis [21]. In studies of chromosomal recessive genetic disorders, the proportion of CD8 + T cells was found to be negatively associated with *IGHG* [22]. This finding was consistent with our analysis, which showed that *IGHG1* was negatively correlated with CD8 + T cells. Structural variants of lymphocytes associated with the *IGHV* gene family [23]. This observation indicates that IGHV1-OR15–9 potentially influences CD8 + T cells, thereby modulating cellular immune responses. In immunological contexts, LILRB1 functions as a negative regulator of human CD8 + effector T cells [24]. while P2RY13 expression exhibits an inverse correlation with CD8 + T cells [25]. *PIPOX* has a significant effect on cell proliferation, growth, and death [26], and we found that *PIPOX* is negatively correlated with CD8 + T cells. Studies on the *RN5S* gene family were conducted in the 1990s and were limited to the genomics of oncogenic adenovirus type 12 infection of human cells. Our study was the first to reveal that RN5S154 and RN5S477 exhibited a significant correlation with CD8 + T cells infiltration. This suggested that further research into the relationship between the *RN5S* gene family and immunization is needed.

In resolving the function and diversity of CD8 + T cells and the molecular mechanisms of immune response, single-cell technology allows us to perform high-throughput sequencing analyses of genomes, transcriptomes, and epigenomes at the level of single cells, which helps to reveal the gene structure and gene expression of individual CD8 + T cells [27]. Integrating the PanglaoDB database and the GSE134649 dataset, it was corroborated that *LILRB1* and *CTL/CTLD* superfamily members are dominated by DCs of activated initial T cells and played a crucial role in CD8 + T cells regulation (Fig 5A), reflecting the heterogeneity of the CD8 + T cells cluster (Fig 5B). This reverse-validated the aforementioned scientific hypotheses at single-cell level in our study.

Reverse validations also come from drug development and applications. Amlodipine (DB00381) and gabapentin (DB00996) are calcium blockers that may theoretically regulate CD8 + T cells infiltration into target tissues by blocking calcium flux, reduce rectal sensitivity to distension, and improve rectal compliance in patients with IBS and lower rectal sensory thresholds [28,29]. Sodium acetate (DB09395) and alpha-linolenic acid (DB00132) are fatty acids that can enhance CD8 + T cells effector functions by improving cellular metabolism and reducing the frequency and amplitude of colonic contractions [30,31]. Technetium Tc-99m tilmanocept (DB09266) is a radiopharmaceutical receptor that recognizes the largest number of diseased cells in many lymph nodes, and its intake is theoretically positively correlated with CD8 + T cells [32]. Copper (DB09130) can be combined with a variety of components to enhance the cytotoxicity of CD8 + T cells, while high erythrocyte copper levels may have a protective effect against the development of IBS [33]. Testosterone (DB00624) reduces the activation of CD8 + T cells, affecting the immune response during the course of the disease, while the level of peripheral testosterone levels in male patients with IBS is significantly different from those in healthy individuals [34,35]. Promethazine (DB01069) improves fibromyalgia pain and mood disorders. Glycine (DB00145) supplementation attenuates the infiltration of CD8 + T cells lymphocytes into the colon and is a nutritional strategy to ameliorate LPS-induced intestinal injury [36,37]. In the behavior of FMT to alleviate anxiety and depression in IBS, KEGG functional analysis confirmed the top five enrichment pathways of the “glycine, serine, and threonine metabolism” pathway [38]. Drug-gene interactions were oriented toward IBS or CD8 + T cells infiltration.

Immune and microbial barriers naturally coexist in the intestine and together maintain intestinal homeostasis [39]. The interaction mechanisms between them have always been a fascinating topic. The advent of FMT has provided a promising strategy for the treatment of intestinal diseases such as IBS and has opened up new perspectives for the study of immunity and microecology. Mainstream research has recognized that adaptive immunity is achieved through the differentiation and maturation of B and T cells, as well as the establishment of immune tolerance to microbiota, while the main focus around the human body remains keen on the design of the RCT scheme. This facilitates the optimization of clinical drug delivery schemes, the enrichment of evidence-based medicine evidence and the introduction of FMT-related guidelines [40,41]. However, this is not conducive to deeply considering the initiating factors and then analyzing their supporting mechanism to overcome various challenges that have arisen in the treatment of IBS with FMT. Further, this prevents a panoramic view of the overall interaction between the microbiota and the host immune response in both healthy and diseased states. In the context of multi-omics, starting from key gene mining analysis, this study effectively integrated multi-dimensional information, including transcriptomics, immune infiltration, single-cell sequencing, and drug development. This process restores the essence of biological events from multi-dimensional fragmented scenes in interactive iterative microbiome exploration. This helped us confirm scientific hypotheses and comprehend the bioinformatic foundation of FMT treatment for IBS from the perspective of CD8 + T cells activation. This also provides theoretical support for future proof-of-concept schemes, innovative FMT clinical programs, and research paradigms orienting CD8 + T cells and their derived factors.

However, this study had several limitations. First, the limited sample size is an inherent limitation of bioinformatics studies based on public databases. GSE138297 is currently the only transcriptome dataset in the GEO database that includes paired colonic biopsy tissues from IBS patients before and after FMT. Such data are extremely scarce and comprise only eight paired samples from IBS patients before and after FMT. However, the conclusions of this study are reliably supported by multi-dimensional biological validations, including functional enrichment, immune infiltration, single-cell expression, and drug-gene interaction analyses. At present, no matching independent validation set is available in public databases, so external validation cannot be conducted. In future studies, we will expand the clinical sample size and conduct independent cohort validation and sensitivity analysis to further verify the conclusions. Furthermore TIMER 2.0 was initially developed for tumor microenvironment analysis. While TIMER 2.0 allows immune cell abundance assessment across different tissue types, the absence of complementary validation with tools specifically optimized for non-neoplastic tissues (e.g., CIBERSORTx) represents a methodological limitation. Existing studies are scattered across the fields of RCT, transcriptomics, microbiomics, and metabolomics. Each of these results provides a detailed interpretation in a certain domain, but systematic integration has been lacking, resulting in a loss of macroscopic comprehension of the mechanisms and limited optimization for FMT programs. By contrast, our study involved tissues, molecules, cells, and drugs, and further integrated multi-dimensional data to decipher and validate the mechanisms of FMT in IBS. Second, we utilized data from healthy individuals for single-cell sequencing to analyze the expression levels of key genes in CD8 + T cells, rather than data from patients with IBS. We found that the available single-cell literature on intestinal diseases has mostly focused on inflammatory bowel disease or intestinal tumors, but has not yet addressed IBS. Obtaining single-cell information on CD8 + T cells in the equilibrium state from healthy individuals could prevent standard deviations toward other diseases. This is beneficial to provide a standard reference for future single-cell research involving IBS.

## Conclusions

Using bioinformatics approaches, we obtained ten key genes associated with the therapeutic process of FMT for IBS. An association between FMT, key gene regulation, and CD8 + T cell activation-related immunoregulation was indicated at different biological levels, such as tissues, molecules, cells, and drugs. This study provided a research paradigm for the theoretical support of similar proof-of-concepts.

## Abbreviations

FMT: fecal microbiota transplantation
IBS: irritable bowel syndrome
DEGs: differentially expressed genes
WGCNA: weighted correlation network analysis
BP: biological process
GSEA: gene set enrichment analysis
RCT: randomized controlled trial
GEO: gene expression omnibus
GO: gene ontology
MM: module membership
GS: gene significance
DCs: dendritic cells
KEGG: kyoto encyclopedia of genes and genomes
